# Is All Hypocalcemia Due to Low Parathyroid Hormone?: An Unusual Case of Pseudohypoparathyroidism in a Young and Healthy Patient

**DOI:** 10.7759/cureus.16243

**Published:** 2021-07-07

**Authors:** Farhan A Shah, Priscilla Fujikawa, Michalla Braford, Kashyap Patel, Kamran Rasul

**Affiliations:** 1 Internal Medicine, Lewis Gale Medical Center, Salem, USA; 2 Internal Medicine, Edward Via College of Osteopathic Medicine, Salem, USA; 3 Endocrinology and Diabetes, Lewis Gale Medical Center, Salem, USA

**Keywords:** hypocalcemia, pseudohypoparathyroidism, parathyroid disorder, endocrinology and diabetes, parathyroid hormone

## Abstract

Pseudohypoparathyroidism is a rare disorder that is characterized by hypocalcemia and hyperphosphatemia that are unresponsive to the parathyroid hormone. We present a unique case of pseudohypoparathyroidism seen in a 33-year-old male who presented with abnormal laboratory findings including hypocalcemia, hypokalemia, hypomagnesemia, hyperphosphatemia, and positive Trousseau's sign.

Pseudohypoparathyroidism is a rare phenomenon, with an estimated prevalence between 0.3 and 1.1 cases per 100,000. Genetic mutations have been noted to play a role in the presentation of pseudohypoparathyroidism. Clinical phenotypical manifestations can include brachydactyly, short stature, a stocky build, and a round face. Similar routine follow-up and treatment as traditional hypoparathyroidism is practiced. This case highlights a unique endocrinologic pathology that clinicians should be more aware of, as it can be confused with hypoparathyroidism.

## Introduction

Pseudohypoparathyroidism (PHP) is a rare phenomenon. The prevalence is not known; however, estimated prevalence varies between 0.3 and 1.1 cases per 100,000 with studies conducted in Denmark suggest a prevalence close to 1.1 per 100,000 [1-3]. PHP was first described by Albright et al. in 1942 after discovering a patient who had elevated phosphate and diminished calcium in the setting of an elevated parathyroid hormone (PTH). Albright first called the phenomenon Sebright-Bantam, after the Sebright bantam chicken, given that a male fowl has similar feathers to the female in the setting of functioning testes [4]. We present a unique case of PHP in a relatively healthy African-American male.

## Case presentation

A 33-year-old African-American male with a past medical history of recently diagnosed hypertension visited his primary medical physician's office for a routine annual visit. The patient was later alerted from his doctor's office that his bloodwork revealed hypocalcemia and needed to go to the emergency department to be evaluated. The patient was not complaining of any symptoms, including fatigue, weakness, or lightheadedness.

In the emergency department, on physical examination, he had a positive Trousseau's sign. His vital signs were only remarkable for an uncontrolled elevated blood pressure of 195/109. His abnormal laboratory findings included potassium 3.1 mmol/L (reference 3.5-5.1 mmol/L), calcium 5.7 mg/dL (reference 8.5-10.1 mg/dL), ionized calcium 0.61 mmol/L (reference 1.15-1.35 mmol/L), phosphorous 5.0 mg/dL (reference 2.6-4.7 mg/dL), and magnesium 1.5 mg/dL (reference 1.8-2.4 mg/dL). He was admitted to the hospital for acute hypocalcemia. 

While hospitalized, the patient was started on daily calcitriol along with Os-Cal 1 g calcium supplementation with each meal t.i.d. His antihypertensives were increased and his other electrolytes were repleted. The patient's intact PTH level was elevated at 280.9 pg/mL (reference range 18.5-88.0 pg/mL), ionized calcium improved to 1.02 mmol/L, and calcium improved to 7.3 mg/dL. The patient was diagnosed with PHP. Subsequently, on day 3 of his hospitalization, the patient was discharged, with a plan to test for the *GNAS1* mutation on an outpatient basis. 

## Discussion

PHP is a unique phenomenon that is characterized by hypocalcemia and hyperphosphatemia where tissues are unresponsive to the action of the PTH [1]. The first cases of PHP were described in individuals with specific developmental morphologies with features such as brachydactyly, cognitive impairment, round faces, heterotropic subcutaneous ossification [5]. This particular set of morphologies were deemed the PHP type 1A (PHP1A) [5]. Later on, Albright et al. identified a similar subset of patients who had many of the same physical features but normal values for calcium, phosphorus and PTH as well as a normal response to PTH. He referred to these individuals as having pseudopseudohypoparathyroidism (PPHP) [5]. The phenotypes for PHP1A as well as the phenotype for PPHP together are known as Albright’s hereditary osteodystrophy (AHO) [5].

Further research and studies have been developed to allow greater understanding and classification of PHP. Broadly speaking, there are two main classifications of PTH, type 1 and type 2. Type 1 is further broken down into three different classifications: PTH type 1a, PTH type 1b, and PTH type 1c [3]. Type 1 demonstrates a depressing cyclic adenosine monophosphate (cAMP) and phosphate excretion compared to PTH where the cAMP response is normal but phosphate excretion is depressed following the binding of PTH to receptors [3]. PHP-related disorders also exist, which involve genetic defects in the Gs pathway such as *PRKAR1A*, *PDE4D*, *PDE3A* or *GNAS* [6].

While there are differences between classifications, individuals may initially present with hormonal resistances with subsequent subclinical hypothyroidism, hypogonadism, type 2 diabetes, limited mobility due to skeletal issues, ectopic ossifications, neurohormonal deficits, short stature, and brachydactyly [6].

The lack of response to PTH is attributed to a mutation in the *GNAS *gene, located on chromosome 20q13.32. The GNAS gene is responsible for the creation of alpha subunits in the heterotrimeric G protein complex and subsequent activation of adenylyl cyclase [2]. Numerous hormones in the body act through a G-protein coupled receptor, including thyroid-stimulating hormone (TSH), follicle-stimulating hormone (FSH), luteinizing hormone (LH), adrenocorticotropic hormone (ACTH), growth hormone-releasing hormone (GHRH), and antidiuretic hormone (ADH). Individuals who have a mutation in the alpha subunit of Gs may also experience states mimicking deficiencies in these pathways [2].

The presentation of individuals with PHP depends heavily on the location of the mutation. Individuals with PHP1A have a variable presentation, depending on whether a mutation in GNAS is present on the maternally or paternally inherited allele. If the mutation is present on the maternally inherited allele, there is multiple Gs protein resistance that lead to resistance to multiple Gs protein-coupled hormones including PTH, TSH, LH, FSH, and GHRH, which is attributed to the paternal imprinting of Gas transcripts [5]. Individuals with this mutation will often have early-onset severe obesity, whereas individuals who have the GNAS mutation on a paternally inherited allele will have the AHO phenotype and no hormonal resistance or obesity [5].

Diagnosis of PHP relies on both clinical and biochemical features. Clinical features that are considered major criteria include brachydactyly type E with premature fusion of the epiphyses and short stature by adulthood compared to the unaffected parent. Additional criteria include stocky build, round face in comparison with siblings, obesity, and ectopic ossifications [7]. However, our patient did not present with this phenotype nor did he have any family history of hypocalcemia. Biochemical major criteria include PTH resistance, which develops gradually in 45% to 80% of patients, subcutaneous ossifications, early-onset obesity with TSH resistance or with any of the other biochemical major criteria, and AHO. Other features that support this diagnosis are elevated TSH, congenital hypothyroidism, hypogonadism, growth hormone deficiency, cognitive/hearing impairment, Chiari malformation type 1, carpal tunnel syndrome, enamel hypoplasia, oligodontia, advanced skeletal maturation, and central nervous system calcifications [6,7].

Upon diagnosis, PTH, calcium and phosphorus levels should be checked every six months in children and every 12 months in adults. Calcium levels should be more closely monitored in patients who are in acute phases of growth, during an illness, pregnancy, or breastfeeding. Calcifediol (vitamin D3 analog) should be maintained as close to the normal level as possible [7].

Treatment is similar to that of hypoparathyroidism and is started when PTH is greater than twice the upper limit of normal regardless of calcium levels. Patients are managed with active vitamin D metabolites, such as calcitriol, and calcium supplementation as needed to maintain normocalcemia. PTH levels should also be maintained as close to normal as possible due to the potential for skeletal demineralization resulting from prolonged exposure to high PTH, potentially leading to tertiary hyperparathyroidism [1,6]. Other aspects of treatment involve the same approach seen with any other hormonal abnormalities. For example, levothyroxine is used for elevated TSH and GH deficiency is treated with recombinant human growth hormone [7].

Annual laboratory exams include PTH, TSH, calcium, phosphorus, and urine calcium. Although the sensitivity to PTH remains intact in the distal renal convoluted tubules, urine calcium should be monitored and those who are persistently hypercalciuric should undergo renal imaging [6]. In children, height should be monitored at least every six months until the final height is achieved. Tanner staging also needs to be monitored to evaluate the appropriate progression of pubertal maturity [7]. Children should also undergo GH deficiency testing in order to initiate treatment in a timely manner [1]. 

Patients affected by PHP are prone to increased risk factors for osteoporosis, but there is no indication for routine dual-energy x-ray absorptiometry measurements due to the lack of proven increased fracture risk [7]. All patients with the *GNAS* mutation should be examined for cutaneous bony plaques, including size, location, joints involved, and triggering events. However, routine imaging of these ossifications is not recommended unless they are symptomatic or under consideration for surgical excision, in which case CT or MRI should be obtained [7].

In a large epidemiological study on PHP, patients with PHP were found to be at a higher risk to develop cataracts, infections, seizures, and neuropsychiatric disorders [2]. The resulting hypocalcemia could cause long QT syndrome and cardiomyopathy [2]. Since calcium plays a significant role in stabilizing the cell membrane, hypocalcemia could result in seizures. Patients with PHP have been observed to have grand mal-like seizures in addition to other types of seizures [8]. Calcium operates as a secondary messenger for neutrophils [9]. Consequently, hypocalcemia in PHP may result in a delayed secondary immune response, increasing the risk of developing infections [2].

## Conclusions

PHP is a rare disorder that can mimic traditional hypoparathyroidism. Cases such as ours help illustrate the laboratory and clinical manifestation of the disease. Further exposure to similar cases will help clinicians become more cognizant of the condition and can help diagnose and treat their patients suffering from the pathology.
